# Correlation between red blood cell distribution width, neutrophil to lymphocyte ratio, and neutrophil to platelet ratio with 3-month prognosis of patients with intracerebral hemorrhage: a retrospective study

**DOI:** 10.1186/s12883-022-02721-2

**Published:** 2022-05-24

**Authors:** Sheida Shaafi, Ehsan Bonakdari, Yalda Sadeghpour, Seyed Aria Nejadghaderi

**Affiliations:** 1 Department of Neurology, School of Medicine, Tabriz University of Medical Sciences, Tabriz, Iran; 2 Neuroscience Research Center, Tabriz University of Medical Sciences, Tabriz, Iran; 3 Research Center for Integrative Medicine in Aging, Aging Research Institute, Tabriz University of Medical Sciences, Tabriz, Iran; 4 School of Medicine, Shahid Beheshti University of Medical Sciences, Koodakyar Ave., Daneshju Blvd., Velenjak, Tehran, Iran

**Keywords:** Red cell distribution width, Intracerebral hemorrhage, Neutrophil to lymphocyte ratio, Neutrophil to platelet ratio, Prognosis

## Abstract

**Background:**

Red cell distribution width (RDW) is a parameter that indsicates the heterogeneity of red blood cell size and could be a prognostic factor in some diseases. Also, intracerebral hemorrhage (ICH) is considered a vascular event with a high mortality rate. We aimed to examine the role of RDW, neutrophil to lymphocyte ratio (NLR), and neutrophil to platelet ratio (NPR) in predicting the prognosis of patients with ICH.

**Methods:**

This is a retrospective cohort study conducted on 140 patients with ICH admitted to the neurology ward and intensive care unit (ICU) in Imam Reza Hospital, Tabriz, Iran. Demographic data, National Institutes of Health Stroke Scale (NIHSS), and complete blood count test parameters were evaluated within 24 h after hospitalization. These variables were collected and re-evaluated three months later.

**Results:**

The mean age of the study population was 61.14 (± 16) years and 51% were male. The mean NLR (*p* = 0.05), neutrophil count (*p*=0.04), platelet count (*p* = 0.05), and NIHSS (*p*<0.01)  had a significant difference between the deceased patients and those who partially recovered after three months. The ROC curve showed that NIHSS (area under curve (AUC): 0.902), followed by NPR (AUC: 0.682) variables had the highest AUC.

**Conclusion:**

RDW could be a relevant prognostic factor and predictor in determining 3-months survival in ICH. Nevertheless, further large-scale prospective cohorts might be needed to evaluate the associations.

**Supplementary Information:**

The online version contains supplementary material available at 10.1186/s12883-022-02721-2.

## Introduction

Red cell distribution width (RDW) is a parameter that represents the dispersion of erythrocyte volume. Coefficient of size changes of red blood cells is more accurate than the structure of red blood cells in peripheral blood smears [1, 2]. The RDW values can be determined easily using an automatic flow cytometer as part of a standard complete blood count (CBC) [3]. Previous studies have shown that abnormal RDW values are highly associated with mortality and poor prognosis [4–6] in various diseases such as acute coronary syndrome, cerebral ischemic disease, heart failure, and peripheral vascular disease [7–9]. Higher RDW values impair blood circulation and oxygen supply, so elevated RDW values are associated with more severe and unfavorable outcomes [10].

Neutrophil to lymphocyte ratio (NLR) and neutrophil to platelet ratio (NPR) are indices that can represent the activities of the inflammatory complex and inflammatory responses in the vascular bed [11]. The NLR is a valuable biomarker for evaluation of inflammatory response following intracerebral hemorrhage (ICH), and it is associated with prognosis, in which the more severe the inflammatory response, the more aggravated nerve injury is expected [12, 13].

In 2019, the total prevalent cases of ICH were 20.66 million (95% uncertainty interval (UI): 18.02–23.42), which showed a 58.0% increase since 1990. Furthermore, ICH was accounted for 68.57 million (95% UI: 63.27–73.68) disability-adjusted life-years in 2019, which increased 25.0% since 1990 [14]. It is also associated with various complications such as hydrocephalus and cerebral edema, which could be even life-threatening [15]. Therefore, early diagnosis and prediction of its prognosis could be helpful for its management in clinical settings.

The predictive value of some blood markers in patients with different types of strokes has been evaluated in the previous research [16]. However, there is a lack of correlation between different CBC parameters simultaneously with prognosis of ICH. Therefore, the aim of this study is to describe the 3-month prognostic utility of RDW, NLR, and NPR in patients with ICH.

## Material and methods

### Study design and subject population

The target population in the present retrospective cohort study was patients with ICH referred to Imam Reza Hospital, Tabriz, Iran, from 2014 to 2019. Using the random sampling method, a total number of 140 participants enrolled in the study. Patients over 18 years old and a definitive diagnosis of first-time cerebral hemorrhage with complete laboratory information were included in the study. Exclusion criteria were age under 18 years, history of cancer, end-stage liver disease, acute or chronic renal failure, receiving blood products in previous three months of hospitalization, ICH following trauma, and long-term duration (i.e., above 48 h) between ICH and hospitalization.

Informed consent was obtained from all patients in the study. The study was approved by the regional ethics committee (Ethics Code: IR.TBZMED.REC.1398.797). All methods were performed in accordance with the national guidelines and regulations.

### Data collection

Patient information, including age, gender, underlying diseases (e.g., hypertension, diabetes, hyperlipidemia, and ischemic heart disease), CBC parameters including white blood cell count, lymphocyte and neutrophil count, hemoglobin concentration, hematocrit, erythrocyte count, mean corpuscular hemoglobin concentration (MCHC), RDW and platelet count, neurological function and severity of the stroke measured using the National Institutes of Health Stroke Scale (NIHSS) score were collected. Moreover, mortality at discharge, within 28 days, and three months later were collected. NLR was calculated by dividing absolute neutrophil count to the absolute lymphocyte count.

### Outcomes

The primary outcome was to determine the relationship between RDW, NLR and NPR with the 3-month prognosis of participants. The secondary outcomes were to determine the RDW, NLR and NPR mean values in patients with ICH. Also, we aimed to evaluate the short-term mortality rate and the effects of using RDW, NLR and NPR simultaneously to predict ICH prognosis.

### Data analysis

Collected data were analyzed by SPSS statistical analysis software version 18 (SPSS Inc., Chicago, IL, USA). First of all, the Smirnov-Kolmogorov test was used to assess the normal distribution of variables. Demographic variables were analyzed by descriptive statistical methods, and presented with percentage, frequency and mean ± standard deviation (SD). Analysis of quantitative variables was performed using independent sample T-test or Mann–Whitney U test. The *p*-values less than 0.05 were considered statistically significant.

## Results

### Baseline characteristics

The average age of 140 patients was 61.14 years with an SD of 16 and ranged from 18 to 96 years old. There were 71 males and 69 females in our sample (Table 1). Hypertension and hyperlipidemia were the most and the least common underlying diseases among the participants, respectively (Table 2). The median (interquartile range [IQR]) of hematoma volume in alive and deceased groups were 10 (5–25) and 35 (13–60) milliliter, respectively. There were 113 supratentorial hematomas, including putamen/internal capsule (*n* = 47), lobar hematoma (*n* = 11), thalamus (*n* = 24), and intraventricular (*n* = 31). In addition, infratentorial hematomas were located in pons (*n* = 15), midbrain (*n* = 1), and cerebellum (*n* = 11).

**Table 1:** Descriptive statistics of numerical variables

	Minimum	Maximum	Mean	Standard deviation
Age(year)	18	96	61.14	16.05
WBC $(\times 1000)$	4.7	32.5	11.63	5.26
Lymphocyte $(\mathrm{\%})$	2.5	52.20	15.70	10.44
Neutrophil ($\%)$	7.20	95.80	77.80	14.92
Hemoglobin (g/dl)	5.70	35.20	13.79	2.71
Hematocrit (%)	19.70	57.40	42.17	5.54
RBC $(\times 1000000)$	2.4	6.80	4.72	0.677
MCHC (g/dl)	16.60	36	32.09	1.93
RDW (%)	11.40	26.6	14.02	2.06
Platelet $(\times 1000)$	103	458	232.860	66.80
NIHSS	0	40	15.86	15.09
NLR	0.78	36.44	8.25	6.29
NPR	0.008	0.78	0.05	0.09

*Abbreviations*: *WBC* white blood cell, *RBC* red blood cell, *MCHC* mean corpuscular hemoglobin concentration, *RDW* red cell distribution width, *NIHSS* National Institutes of Health Stroke Scale, *NLR* neutrophil to lymphocyte ratio, *NPR* neutrophil to platelet ratio

**Table 2:** Descriptive statistics of categorical variables

Underlying disease		Number (%)		
		Both sexes (*n* = 140)	Males (*n* = 71)	Females (*n* = 69)
Diabetes	Yes	23 (16.4)	9 (12.7)	14 (20.3)
	No	117 (83.6)	62 (87.3)	55 (79.7)
Hyperlipidemia	Yes	7 (5.0)	3 (4.2)	4 (5.8)
	No	133 (95.0)	68 (95.8)	65 (94.2)
Hypertension	Yes	106 (75.7)	48 (67.6)	58 (84.0)
	No	34 (24.3)	23 (32.4)	11 (16.0)
Coronary artery disease	Yes	25 (17.9)	15 (21.1)	10 (14.5)
	No	115 (82.1)	56 (78.9)	59 (85.5)

### Outcomes

**Fig. 1:**
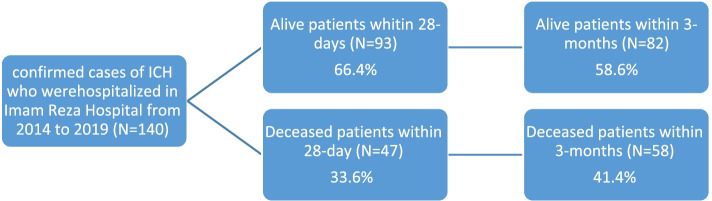
28-day and 3-month outcomes of the study

In this study, the mortality of patients in a short period of 28-day hospitalization and 3 months was considered. Results of mortality study in two time periods showed that in the final result of 3 months, 11 people were added to the number of patients who died in comparison with the 28-day study period and the deceased participants increased from 33.6% to 41.4% (Fig. 1).

### Predictors of 3-month prognosis of patients with ICH

To investigate the effect of the variables on the mortality of patients, the mean of the variables by patients' outcome (death or recovery) was assessed and compared with a T-test between the two groups. Higher NIHSS scores were statistically significant with death within three months (*p* < 0.01), while the higher neutrophil count was associated with higher survival (*p* = 0.04) (Table 3 and Figure S1).

**Table 3:** Comparison of the mean of research variables by 3-month prognosis of patients

Variables	3-month prognosis	Number	Mean	Standard deviation	*P* (t-test)
NLR	Dead	57	9.43	7.10	0.05
	Alive	83	7.44	5.56	
NPR	Dead	57	0.06	0.087	0.41
	Alive	83	0.05	0.093	
RDW	Dead	54	14.37	2.18	0.10
	Alive	80	13.78	1.95	
NIHSS	Dead	57	27.56	14.23	0.00
	Alive	83	7.83	9.33	
Platelet	Dead	57	219.64	57.79	0.05
	Alive	83	241.93	71.27	
MCHC	Dead	57	32.14	1.50	0.80
	Alive	83	32.05	2.18	
RBC	Dead	56	4.60	0.82	0.07
	Alive	81	4.81	0.54	
WBC	Dead	57	12.42	5.13	0.13
	Alive	83	11.08	5.30	
Lymphocyte	Dead	57	13.85	9.98	0.08
	Alive	83	16.97	10.62	
Neutrophil	Dead	57	80.70	11.85	0.04
	Alive	83	75.81	16.47	
Hemoglobin	Dead	57	13.48	2.45	0.25
	Alive	83	14.01	2.87	
Hematocrit	Dead	57	41.78	6.96	0.5
	Alive	83	42.45	4.33	

*Abbreviations WBC* white blood cell, *RBC* red blood cell, *MCHC* mean corpuscular hemoglobin concentration, *RDW* red cell distribution width, *NIHSS* National Institutes of Health Stroke Scale, *NLR* neutrophil to lymphocyte ratio, *NPR* neutrophil to platelet ratio

### Mortality predictors of patients with ICH

**Fig. 2:**
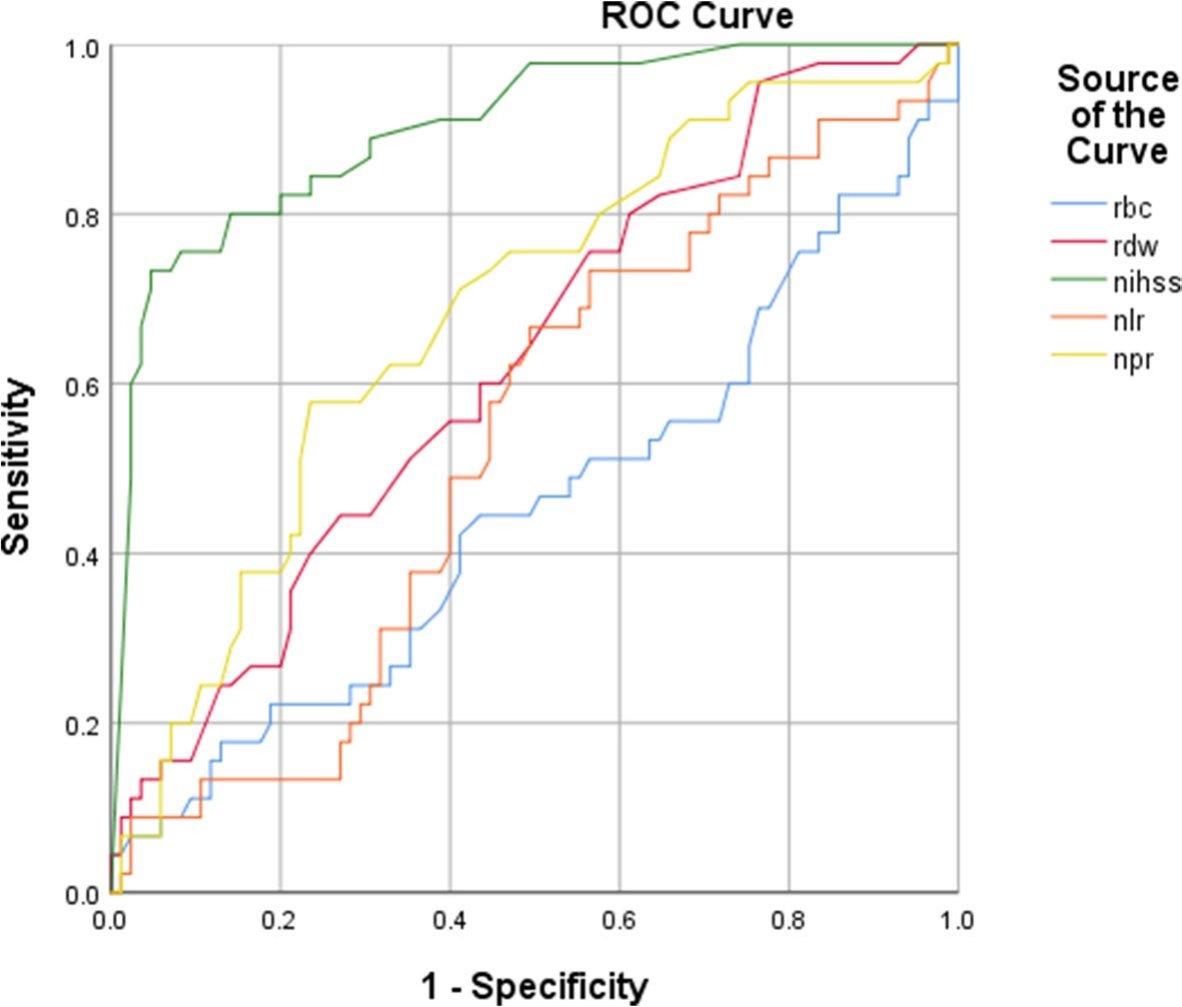
ROC curve of research variables to predict the death of patients at the end of the 3-month period. Abbreviations: RBC: red blood cell; RDW: red cell distribution width; NIHSS: National Institutes of Health Stroke Scale; NLR: neutrophil to lymphocyte ratio; NPR: neutrophil to platelet ratio

The receiver operating characteristic (ROC) curve was utilized to compare the predictions of deaths by variables, including RBC, NIHSS, NPR, NLR, and RDW at the end of the 3-month follow-up. We found that the NIHSS (area under curve (AUC): 0.902), followed by NPR (AUC: 0.682) variables had the highest AUC (Fig. 2 and Table 4).

**Table 4:** Level below ROC diagram for research variables

Tested variables	Levels below the chart
RBC	0.452
RDW	0.627
NIHSS	0.902
NLR	0.535
NPR	0.682

*Abbreviations RBC* red blood cell, *RDW* red cell distribution width, *NIHSS* National Institutes of Health Stroke Scale, *NLR* neutrophil to lymphocyte ratio, *NPR* neutrophil to platelet ratio

## Discussion

The findings of the present study showed that higher NIHSS score and lower neutrophil count are associated with death and NPR could be a mortality predictor in patients with ICH.

RDW as an available laboratory factor, which is a marker of the variation in the volume of red blood cells [3], can be used as a prognostic factor in various health conditions [17]. Moreover, it is associated with chronic inflammation and oxidative stress [5]. Thus, it is reasonable that high RDW values can be known as an integrated risk factor for sub-optimal general health conditions, which is accompanied by a reduction in correction, improvement, and systemic defense capability [5, 9]. In our study, the role of the RDW factor in the prognosis and mortality of stroke patients with ICH in a period of three months was assessed, and it was shown that the RDW value is significantly higher in deceased patients compared to alive patients with ICH. The results of the study conducted by Lorente et al. among 117 patients with spontaneous ICH showed that RDW during the first week was higher in deceased than surviving patients (p ≤ 0.001), which is in accordance with our findings [18]. Furthermore, the article by Kaya et al. conducted on 153 participants, concluded that RDW is an important hematopoiesis indicator for the occurrence of stroke in patients with heart failure, which reveals that high RDW is associated with the development of stroke and is strongly associated with cardiovascular causes [10]. A study by Moreno et al. also showed that RDW is a strong indicator for stroke [19]. Moreover, another study by Kara et al. concluded that RDW, an easily accessible and inexpensive test, is potentially an important parameter in diagnosing stroke and may predict prognosis. Also, in this study NIHSS was used to determine the severity of the stroke and it was found that the increase in stroke severity is associated with higher RDW, and RDW is a strong predictor of the stroke severity [20].

Of note, a wide range of diseases and even physiological conditions lead to changes in the level of RDW in the human body. For example, different types of anemia, inflammatory diseases, and cancers can cause changes in the size of red blood cells [21]. Thus, RDW may not be specific enough to screen for stroke in many patients. One of the most appropriate scales to assess the stroke severity is NIHSS which measures the severity of a neurological deficit. The NIHSS is a standard diagnostic tool for quickly assessing the severity of a stroke and related neurologic deficits, which is used in determining the prognosis. NIHSS had the highest association with the mortality rate in stroke patients, so this variable was significantly higher among the deceased group. Also, the area under the ROC curve for this variable shows the expressive power of predicting the death of patients by this variable [22]. In addition to RDW and NIHSS, NLR, platelets, and neutrophils were significantly effective in determining the prognosis of the intracerebral hemorrhage [23].

The NLR is calculated as dividing the number of neutrophils to the number of lymphocytes in peripheral blood and measures the inflammatory response [24]. An acute inflammatory response occurs in severe conditions such as ischemic stroke, cerebral hemorrhage, and acute coronary syndrome. Therefore, this marker can determine the prognosis and evaluation of disease outcomes in the mentioned diseases [11, 24, 25]. In this regard, our results also showed that higher RDW, NIHSS, RBC, and platelet count increases the risk of short-term and long-term mortality in patients with stroke.

Numerous studies have shown that high levels of neutrophils are sensitive markers for determining mortality [23]. Jickling et al. and Maestrini et al. showed that high neutrophil level is associated with high infarct volume and poor prognosis [26, 27]. In addition to its function in homeostasis, platelets also play a role in the inflammatory responses. In fact, platelets can facilitate neutrophil entry into the brain parenchyma, and higher neutrophil counts will lead to more severe damage [28].

We acknowledge that our study had several limitations. First, since it was a retrospective study, restrictions in data collection, participant inclusion, and lack of a control group exist. Second, it was conducted in a single hospital. Third, the variables were only measured in two time points in 28-days, and 3-months and they were not remeasured during the period of hospitalization. Fourth, the follow-up was limited to 3 months and no long-term follow-up was conducted.

## Conclusions

The current study demonstrated that NIHSS and neutrophil count are associated with ICH mortality. According to the results of the present study, it is suggested to increase the sample size in future studies to increase the accuracy of the results, control and delete other probable variables affecting RDW in the sample, including the information about the region and volume of hemorrhage and evaluate the relationship between study variables and the prognosis of patients in different age groups.

## Supplementary Information

**Additional file 1: Figure S1.** Evaluation of mean variables by patient's final prognosis.

## Data Availability

The datasets generated and/or analyzed during the current study are not publicly available due for they are personal data but are available from the corresponding author on reasonable request.
