# Supplementary material for: Correlation between red blood cell distribution width, neutrophil to lymphocyte ratio, and neutrophil to platelet ratio with 3-month prognosis of patients with intracerebral hemorrhage: a retrospective study
Source: BMC Neurol. 2022 May 24;22:191. doi: 10.1186/s12883-022-02721-2 (PMC9128218; doi:10.1186/s12883-022-02721-2)
Supplement: Supplementary file 1 — Additional file 1: Figure S1. Evaluation of mean variables by patient's final prognosis. [file 12883_2022_2721_MOESM1_ESM.docx]

Figure S1. Evaluation of mean variables by patient's final prognosis

Abbreviations: WBC: white blood cell; RBC: red blood cell; MCHC: mean corpuscular hemoglobin concentration; RDW: red cell distribution width; NIHSS: National Institutes of Health Stroke Scale; NLR: neutrophil to lymphocyte ratio; NPR: neutrophil to platelet ratio.
